# Supplementary material for: Genetic Variability of Bovine Leukemia Virus: Evidence of Dual Infection, Recombination and Quasi-Species
Source: Pathogens. 2024 Feb 15;13(2):178. doi: 10.3390/pathogens13020178 (PMC10893129; doi:10.3390/pathogens13020178)
Supplement: Supplementary file 1 [file pathogens-13-00178-s001.zip › Supplementary materials.pdf]

Supplementary Materials

Table S1. Sequences of primers used to amplify, sequence and clone the BLV env-gp51 region.

Fragment of BLV Genome	Name	Location	Sequence	CG%	Tm °C *	Ta °C **	Cross-dimers in PCR***
env- gp51	Primers used for first round PCR						
	Amplification 1017 bp fragment						
	P4796	4796–4817[”]	5′- CCTGGCGTTTGCTGAAAGCCTT-3′	54.55	62.1	62.0	Max 3' annealing score (best < 9): 6<br>Max 5' annealing score (best < 15): 11
	P5791	5791–5812	5′-TGTCTGCCCTTAGCCATCAGAG-3′	54.55	62.1		
	Primers used for second round PCR						
	Amplification 923 bp fragment						
	P4833	4833–4852[”]	5′-CGACGGTCCCGAAGACGCCC-3′	75.00	67.6	66.0	Max 3' annealing score (best < 9): 6<br>Max 5' annealing score (best < 15): 11
	P5734	5734–5755	5′-CGGCCCTGACCTTAGGCCTAGC-3′	68.18	67.7		

[”]Numbering of genomic locations is based on the first published complete BLV genome (Sagata et al., 1985; GenBank accession no. K02120). * Tm - melting temperature; ** Ta - annealing temperature. The optimal annealing temperature was estabilished using gradient PCR (start with temperature 60 °C and increased up to the extension temperature 72°C). *** Forward / reverse primer pairs cannot form cross-dimers in PCR. Thermo Scientific DyNAzyme II DNA Polymerase, used for nested PCR, is recombinant thermostable DNA polymerase with an improved thermal stability at high temperatures. The polymerase mutation rate is 2.28 x 10-5 mutations per base pair per template duplication. After 35 cycles of PCR amplifying a 804 bp template, 64,16% of the product DNA molecules may have contained 1 (nucleotide) error each. This means that 35,8% of the product molecules may have been entirely error-free. Considering two PCR reactions of 35 cycles each performed with this polymerase, every product molecule contained an averaged 1.28 errors. The next calculation is as follows: 20 clones 1S (or 20 clones 4T) x 1.28 errors = 25.6 errors (nt); 804bp x 20 clones = 16080nt; 16080nt = 100% identity; 25.6 nt = 0.1592%. Mean genetic distance among 20 clones was 2.0% (range: 0.1% - 4.5%) and 2.1% (range: 0% - 4.7%) for isolates 1S and 4T. Based on the above polymerase fidelity calculations, we concluded that this polymerase was suitable for studying the variation of the BLV isolates described in this study.

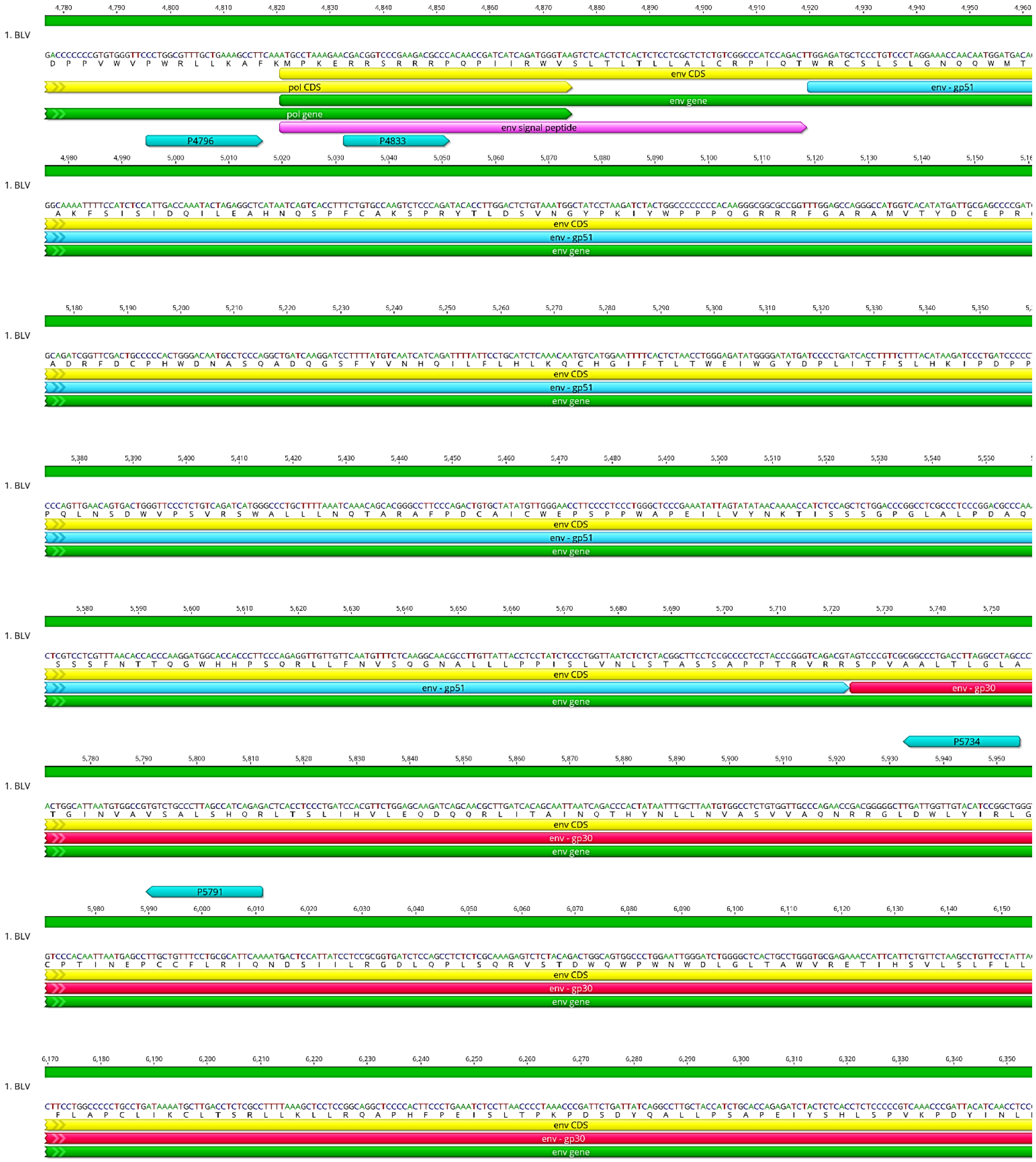

Supplementary Figure 1. Localization of the P4796, P5791, P4833 and P5734 primers on the BLV provirus sequence.

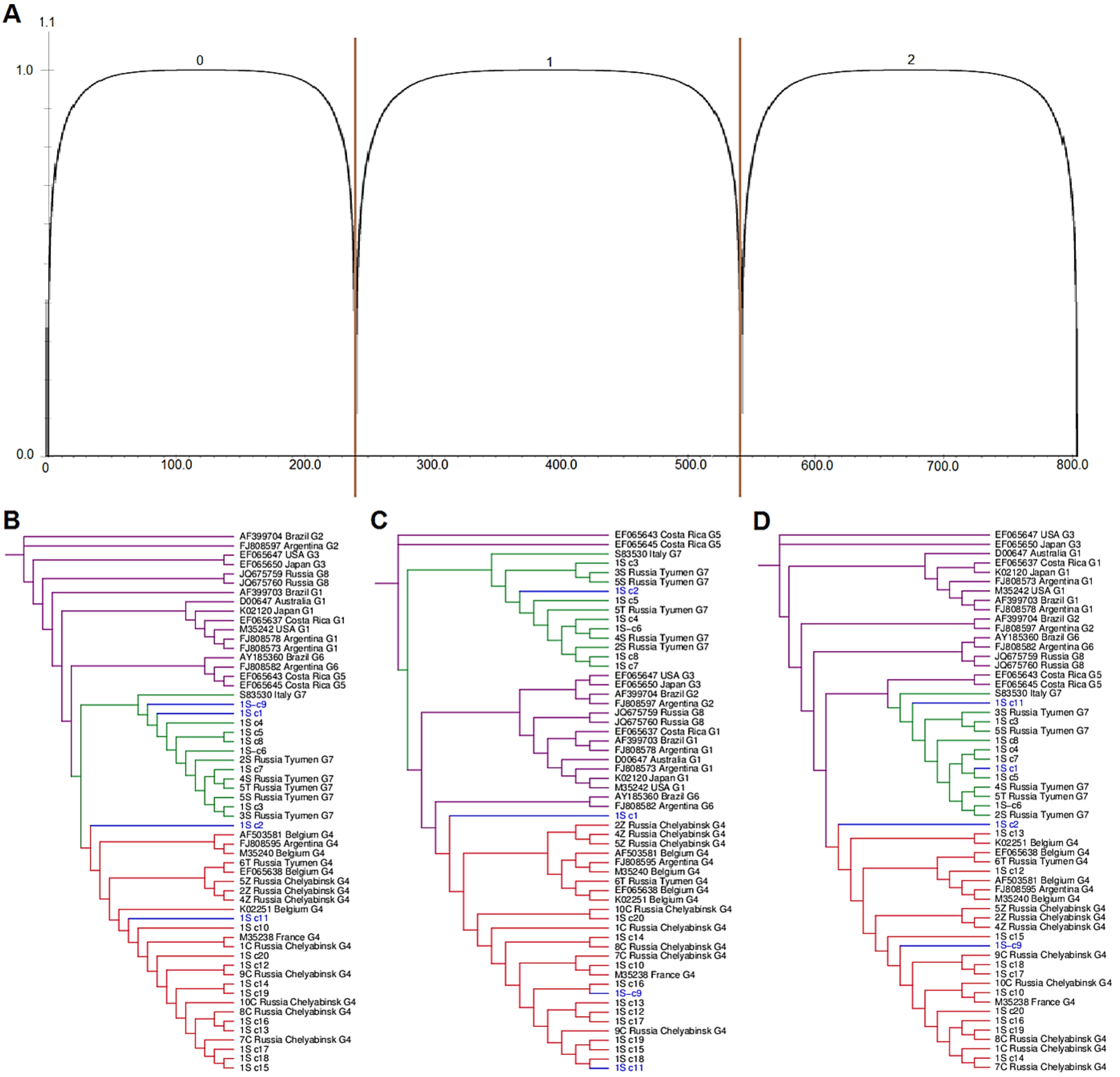

Figure S2: Detection of recombination events for 1S isolate. Phylo-HMM identified two recombination positions. Bio Neighbour-joining phylogenetic trees constructed in HMM for each part of the alignment reported four putative recombinants. A- Two breakpoints predicted at positions 240 bp and 542 bp; Three separate BIONJ trees based on the different portions of the alignment identified these sequences resulted from recombination: 1S-c11 (G4/G4/G7), 1Sc-9 (G7/G4/G4), 1S-c2 (G4/G7/G7) and 1S-c1(G7/G4/G7). B- BIONJ tree based on sequences beginning from 1 to 240 bp of the gp51; C- BIONJ tree based on sequences ranging from 241 to 542 bp of the gp51; D- BIONJ tree based on sequences ranging from 543 to 804 bp of the gp51.

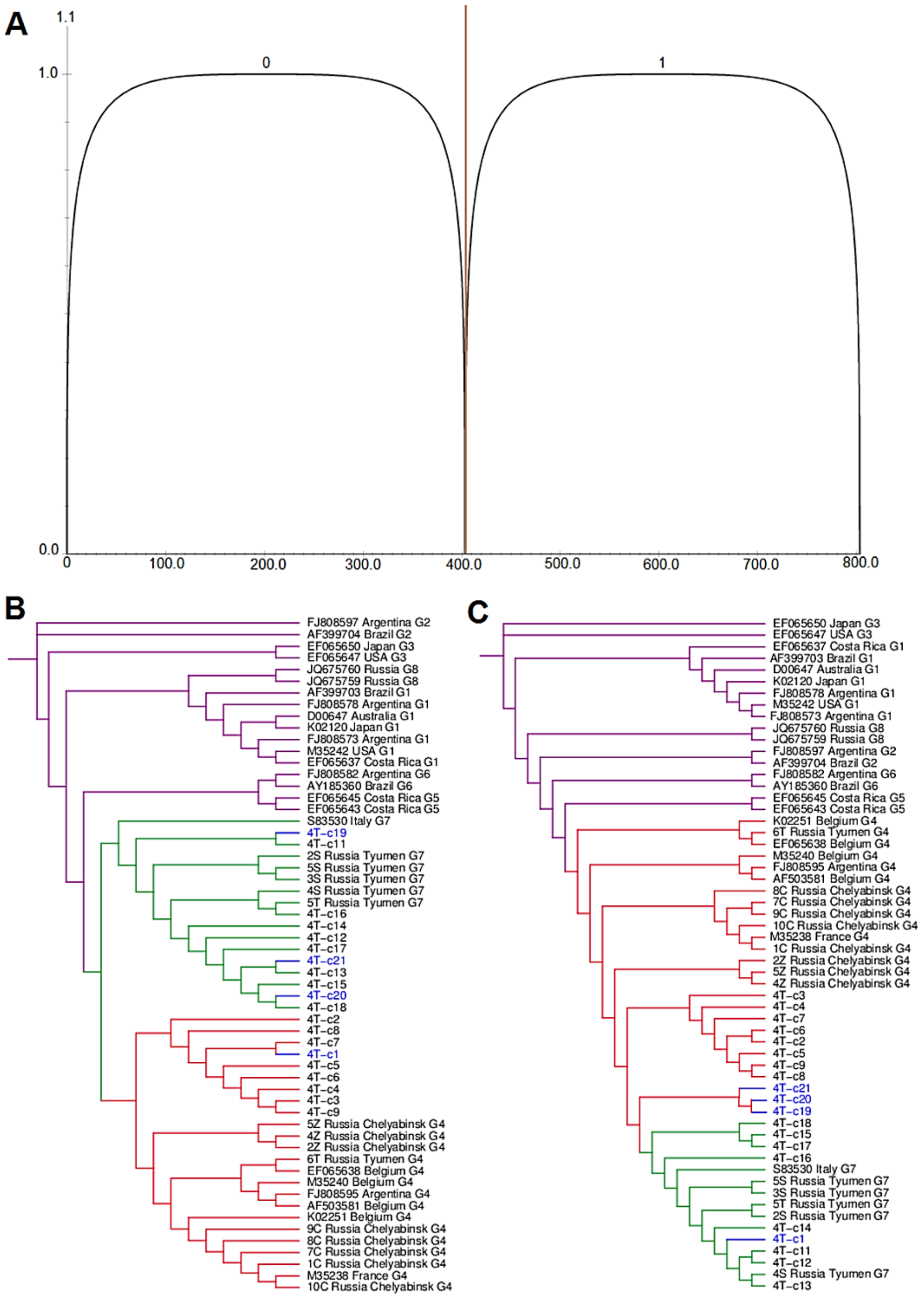

Figure S3: Identification of recombination events for 4T isolate. Phylo-HMM identified one recombination position. Bio Neighbour-joining phylogenetic trees constructed in HMM for each part of the alignment reported four putative recombinants 4T-c1, 4T-c19, 4T-c20 and 4T-c21 marked in blue. A- One breakpoint predicted at positions 400 bp; B- BIONJ tree based on sequences beginning from 1 to 400 bp of the gp51; C- BIONJ tree based on sequences ranging from 401 to 804 bp of the gp51.

A

	42	45	46	76	108	115	118	171	183	192	258	263	288	297	309	327	342	353	366	375	399	576	654	666	741	773	792	799	804
G4 (4T-c8)	G	A	C	A	T	C	C	T	A	G	G	A	T	A	G	T	A	G	C	A	T	A	C	T	G	C	T	A	C
**Recomb. (4T-c20)**	A	C	T	G	A	T	G	C	G	A	A	G	C	G	A	C	G	A	T	G	C	A	C	T	G	C	T	A	C
G7 (4T-c14)	A	C	T	G	A	T	G	C	G	A	A	G	C	G	A	C	G	A	T	G	C	G	T	C	A	T	C	C	T

B

	42	45	46	76	108	115	148	171	183	192	258	263	288	297	309	327	342	353	366	375	399	576	654	666	741	773	792	799	804
G4 (4T-c8)	G	A	C	A	T	C	A	T	A	G	G	A	T	A	G	T	A	G	C	A	T	A	C	T	G	C	T	A	C
**Recomb. (4T-c19)**	A	**A**	T	**A**	**T**	T	G	C	G	A	A	G	C	G	A	C	G	A	T	G	C	A	C	T	G	C	T	A	C
G7 (4T-c14)	A	C	T	G	A	T	G	C	G	A	A	G	C	G	A	C	G	A	T	G	C	G	T	C	A	T	C	C	T

C

	42	45	46	76	108	115	118	171	183	192	258	263	288	297	309	327	342	353	366	375	399	576	654	666	741	773	792	799	804
G4 (4T-c8)	G	A	C	A	T	C	C	T	A	G	G	A	T	A	G	T	A	G	C	A	T	A	C	T	G	C	T	A	C
**Recomb. (4T-c21)**	A	C	T	G	A	T	G	C	G	A	A	G	C	G	A	C	G	A	T	G	C	A	C	T	G	C	T	A	C
G7 (4T-c14)	A	C	T	G	A	T	G	C	G	A	A	G	C	G	A	C	G	A	T	G	C	G	T	C	A	T	C	C	T

D

	45	76	108	118	171	176	183	192	263	288	292	309	327	342	353	359	375	399	424	438	453	460	550	576	654	666	741	773	792	799	804
G4 (4T-c9)	A	A	T	C	T	C	A	G	A	T	C	G	T	A	G	A	A	T	G	T	T	C	A	A	C	T	G	C	T	A	C
**Recomb.(4T-c1)**	A	A	T	C	T	C	A	G	A	T	**T**	G	T	A	G	A	A	T	A	C	C	T	G	G	T	C	A	T	C	C	T
G7 (4T-c13)	C	G	A	G	C	T	G	A	G	C	T	A	C	G	A	T	G	C	A	C	C	T	G	G	T	C	A	T	C	C	T

E

	45	68	90	102	118	162	168	171	183	192	263	270	309	327	332	375	399	460	516	537	576	588	654	666	741	773	799	804
G4 (1S-c14)	A	T	A	T	C	T	C	T	A	G	A	C	G	T	C	A	T	C	A	C	A	T	C	T	G	T	A	C
**Recomb.(1S-c11)**	A	T	A	T	C	**C**	C	T	A	G	A	C	G	T	C	A	T	C	G	T	**A**	**T**	T	C	A	C	C	T
G7 (1S-c7)	C	C	G	C	G	T	T	C	G	A	G	T	A	C	T	G	C	T	G	T	G	C	T	C	A	C	C	T

F

	26	45	68	82	90	118	171	183	192	263	309	327	332	375	388	460	588	654	666	741	773	799	804
G4 (1S-c15)	G	A	T	C	A	C	T	A	G	A	G	T	C	A	T	C	T	C	T	G	C	A	C
**Recomb.(1S-c1)**	A	C	C	**C**	G	G	C	G	A	A	G	T	C	A	**C**	**T**	C	T	C	A	T	C	T
G7 (1S-c3)	A	C	C	G	G	G	C	G	A	G	A	C	T	G	T	C	C	T	C	A	T	C	T

G

	45	68	71	73	90	118	171	208	263	309	327	332	368	375	399	460	576	588	654	666	741	773	804
G4 (1S-c15)	A	T	T	T	A	C	T	A	A	G	T	C	T	A	T	C	A	T	C	T	G	C	C
**Recomb.(1S-c2)**	A	T	T	T	A	C	**C**	**G**	G	A	C	T	**T**	G	C	T	G	C	T	C	A	**C**	**C**
G7 (1S-c8)	C	C	C	C	G	G	C	A	G	A	C	T	A	G	C	T	G	C	T	C	A	T	T

H

	45	68	90	118	171	183	192	206	263	309	327	332	375	399	404	460	482	525	576	588	654	666	799	804
G4 (1S-c13)	A	T	A	C	T	A	G	T	A	G	T	C	A	T	T	C	C	C	A	T	C	T	A	C
**Recomb.(1S-c9)**	C	C	G	G	C	**A**	**G**	C	A	G	T	C	A	T	T	C	C	C	A	T	C	T	A	C
G7 (1S-c6)	C	C	G	G	C	G	A	C	G	A	C	T	G	C	C	T	A	T	G	C	T	C	C	T

Figure S4: Genotype specific nucleotides, on the different fragments manually designated and individually for each recombinant breakpoints confirming the breakpoints calculated by the Phylo-HMM algorithm. A- recombinant 4T-c20; B- recombinant 4T-c19; C- recombinant 4T-c21; D- recombinant 4T-c1; E- recombinant 1S-c11; F- recombinant 1S-c1; G- recombinant 1S-c2 and H- recombinant 1S-c9.

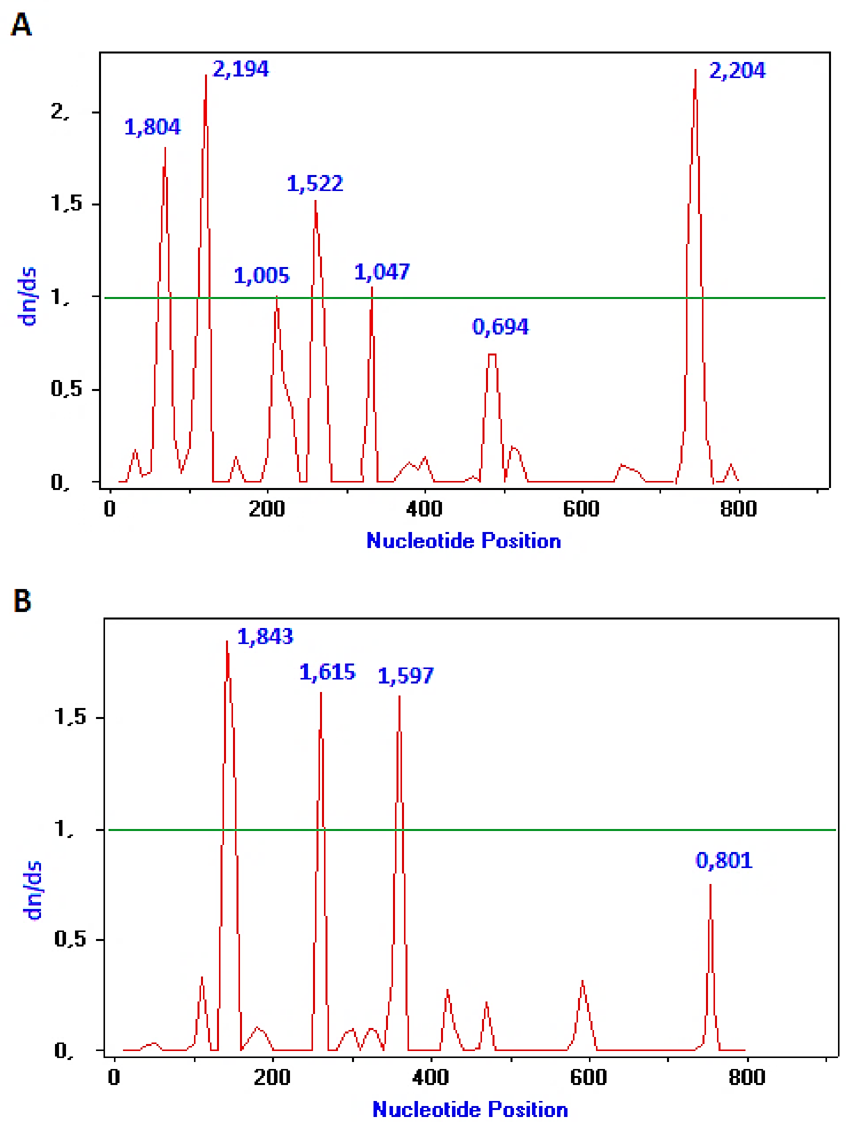

Figure S5. Positive selection among analysed clones within *env* gene coding gp51 protein. The dN/dS ratio is defined as the ratio of nonsynonymous substitutions per nonsynonymous site (dN) to the number of synonymous substitutions per synonymous site (dS). A dN/dS ratio of less than one implies purifying selection, while a ratio of more than one indicates positive selection. A ratio of approximately one indicates a neutral mutation. Sliding window option: Window length: 20, Step size: 10; Units: Coding positions. The red line describes the level of dN/dS for all the windows positioned along the entire length of gp51 (804bp). The blue colour describes the positive selection values. **A** - Positive selection among 20 clones 1S within gp51 was indicated in the following windows: 61-80, 111-130, 201-220, 251-270, 331-350 and 761-780. **B** - Positive selection among 20 clones 4T within gp51 was calculated in the windows: 131-150, 251-270 and 351-370.

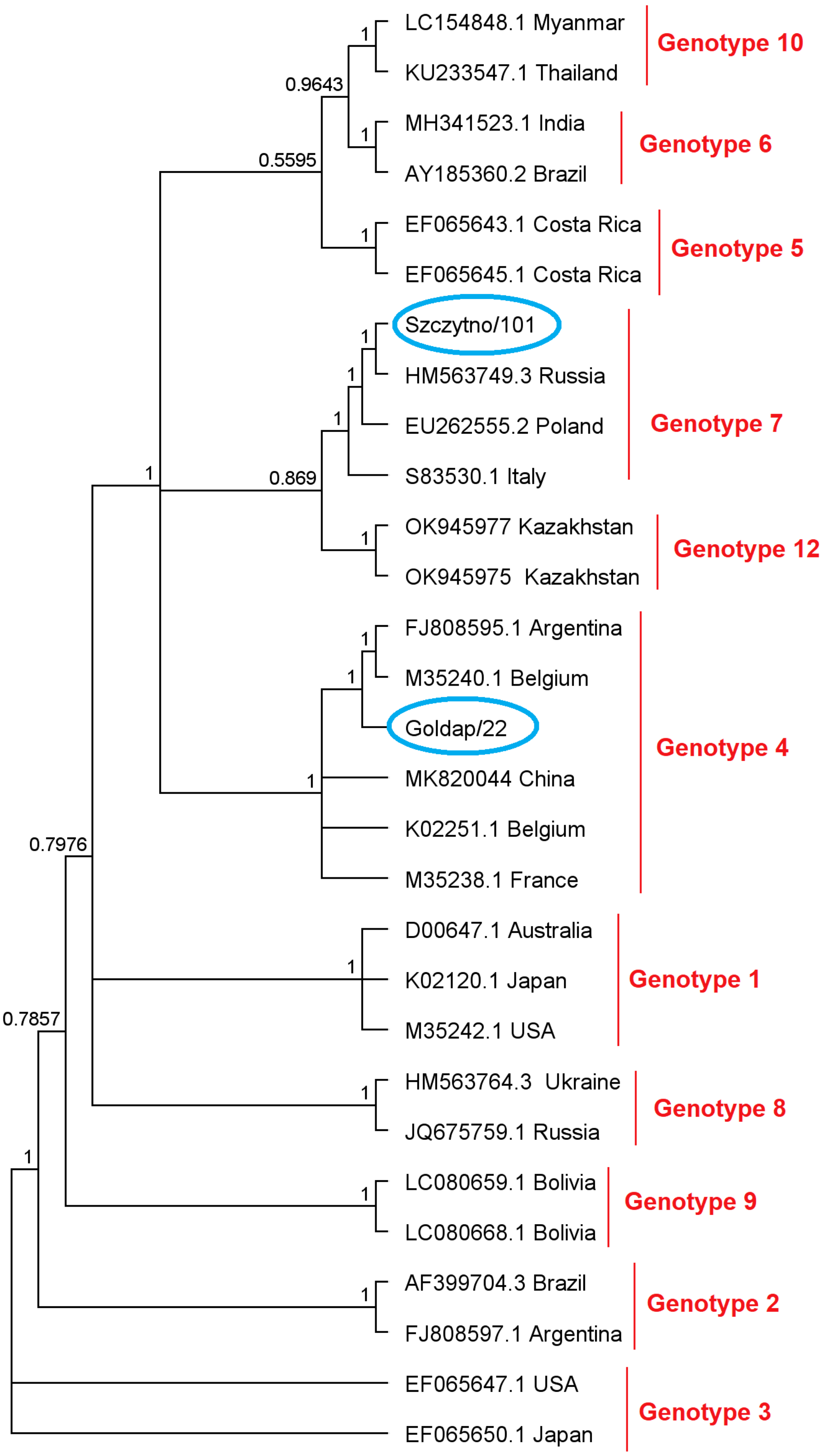

Supplementary Figure 8. Phylogenetic relationship of BLV genotypes. The relationships between sequence Goldap/22 and Szczytno/101 isolated from Polish BLV-infected cattle representing genotypes G4 and G7 (indicated by blue circle, n=2) and additional reference sequences in GenBank (n = 27) were inferred by Bayesian analysis of *env*-gp51 sequences, based on the HKY85 substitution model. The genotypes are indicated at the right by red vertical lines.

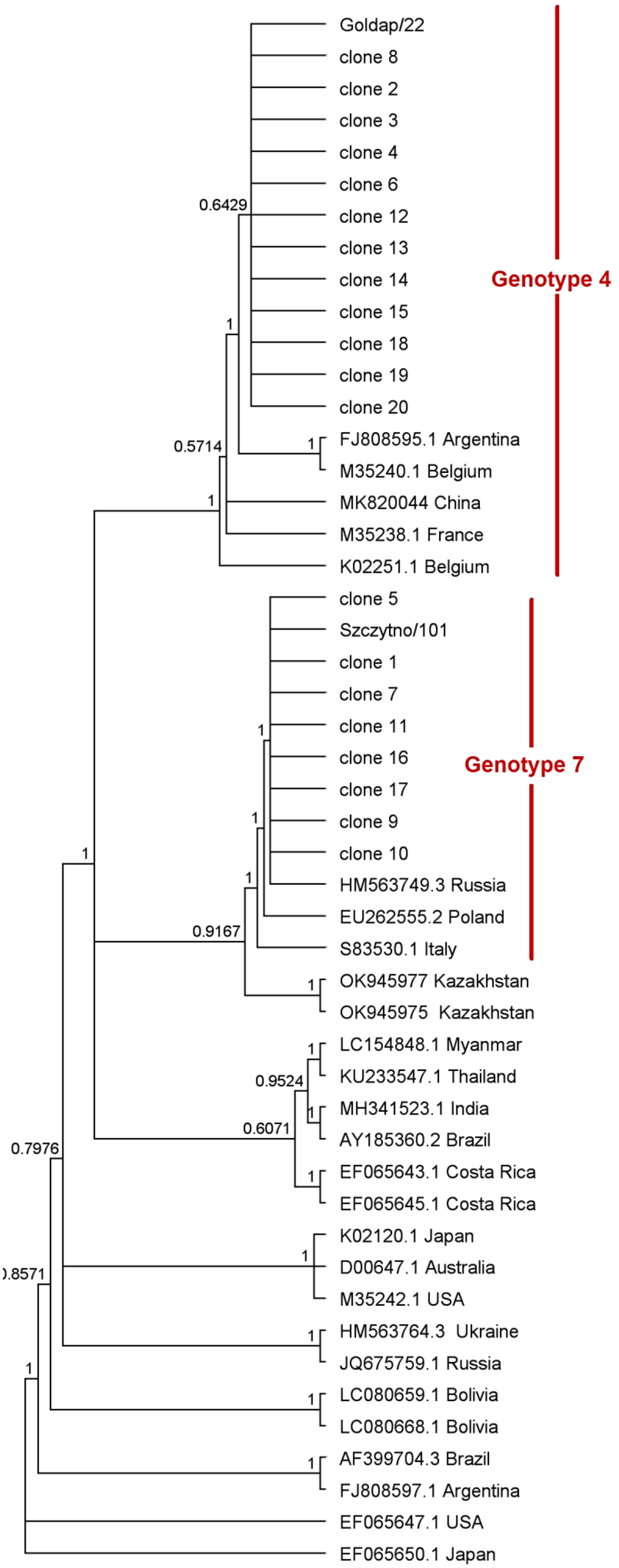

Figure S10: Phylogenetic tree created using Bayes calculations, showing the clustering and distribution of 20 clones (named sequentially from clone 1 to clone 20) derived from a mixture of PCR product containing Goldap/22 (G4) and Sczytno/101 (G7) and sequences representing known BLV genotypes.
