# Supplementary figures and images for: Genetic Variability of Bovine Leukemia Virus: Evidence of Dual Infection, Recombination and Quasi-Species
Source: Pathogens. 2024 Feb 15;13(2):178. doi: 10.3390/pathogens13020178 (PMC10893129; doi:10.3390/pathogens13020178)

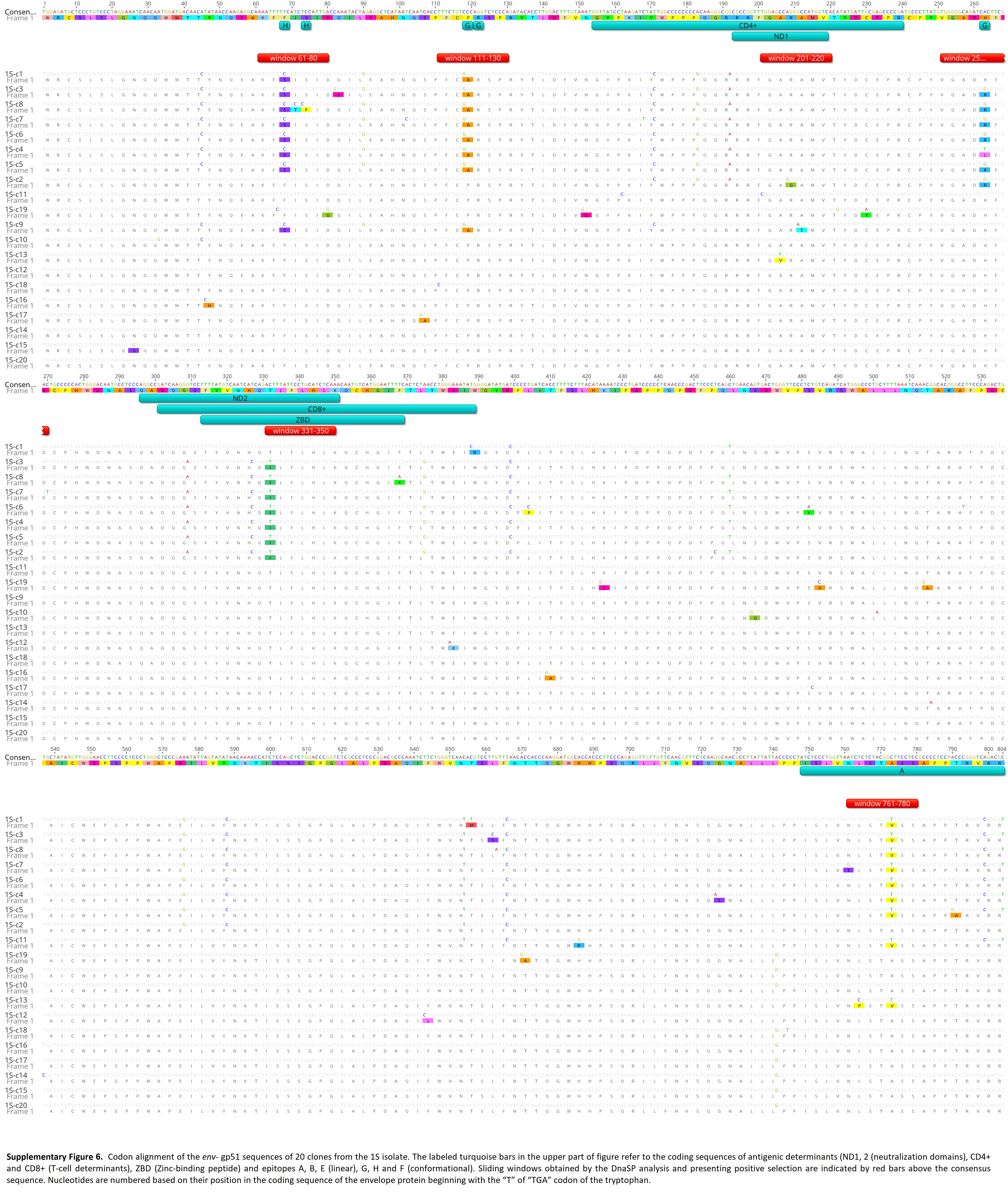

Supplement: Supplementary file 1 [file pathogens-13-00178-s001.zip › Supplementary Figure S6.jpg]

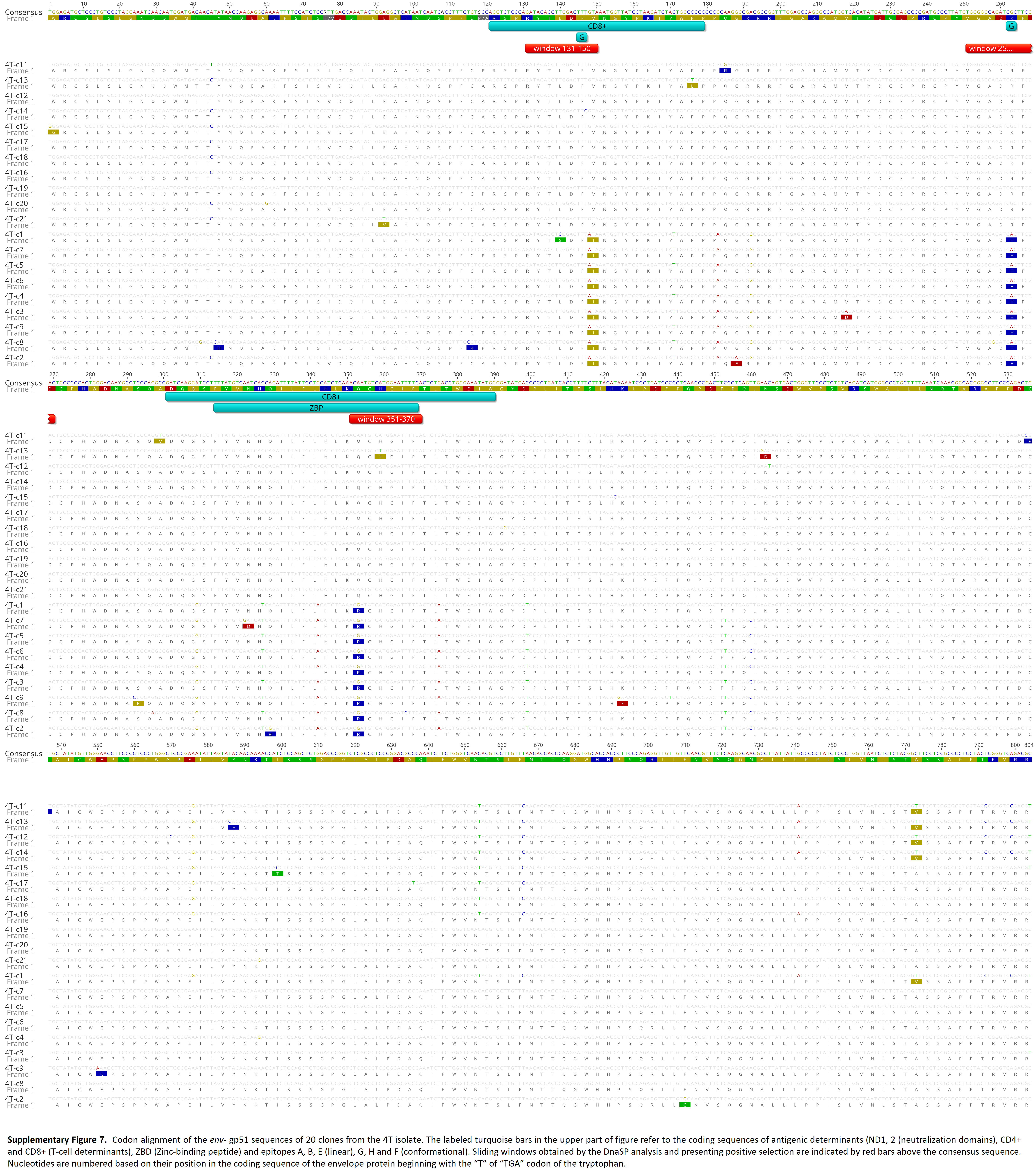

Supplement: Supplementary file 1 [file pathogens-13-00178-s001.zip › Supplementary Figure S7.jpg]

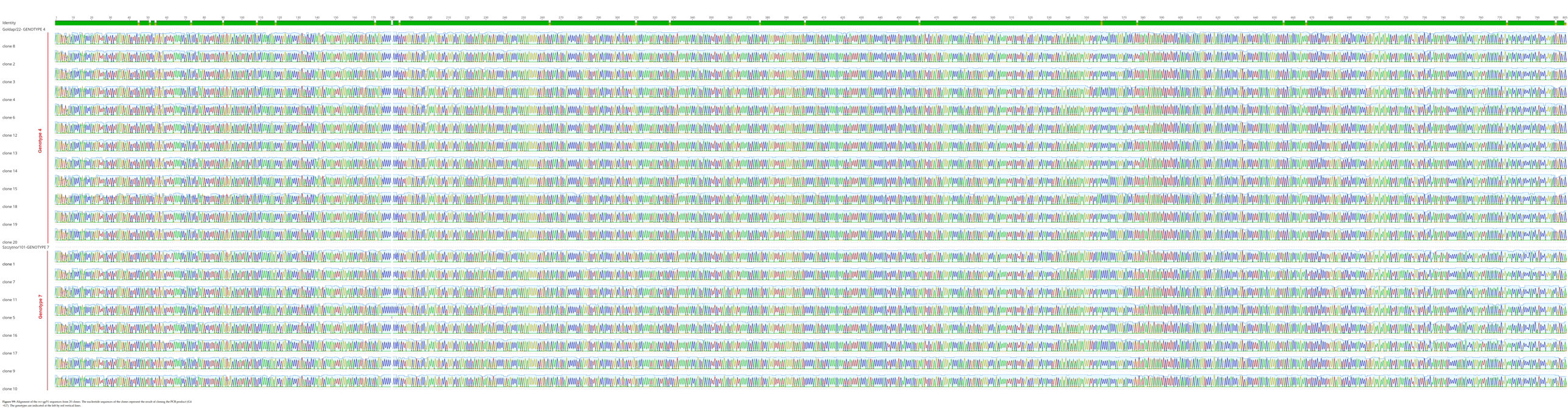

Supplement: Supplementary file 1 [file pathogens-13-00178-s001.zip › Supplementary Figure S9.jpg]
